# The Effect of English as a Foreign Language Teachers’ Optimism and Affectivity on Their Psychological Well-Being

**DOI:** 10.3389/fpsyg.2021.816204

**Published:** 2021-12-23

**Authors:** Xuena Zhang

**Affiliations:** College of Foreign Languages, Henan Institute of Science and Technology, Xinxiang, China

**Keywords:** affectivity, EFL teachers’ optimism, teacher psychological well-being, positive psychology, social principle, positive thinking

## Abstract

Psychological well-being is considered a key component for the mental and physical health of individuals that is influenced by various attributive factors. Some of the important attributes that have a constructive and encouraging effect on the improvement and progress of good habits, positive thinking, behavior, and well-being of individuals like teachers are emotions. Educators’ emotions and emotive features have essential roles in educational circumstances as they affect nearly all facets of their occupation. Moreover, optimism as a new concept is changing from the inspection on positive psychology, social principle, and communal school possessions in education. The present review surveyed the role that affectivity and optimism have regarding the psychological well-being of EFL teachers. In brief, the implications for educators, school managers, teacher-trainers, and forthcoming researchers are provided.

## Introduction

One of the key factors in a learners’ performance success or failure is the cognitive, emotional, and behavioral dimensions of the educators who are directly associated with the learners in various courses to seek a theoretical basis in the field of psychology (Safari and Soleimani, 2019; Xie and Derakhshan, 2021). An emotional setting has been suggested to offer well-being to the entire school community (managers, faculty, learners, and parents) to ensure a healthier setting and facilitate both education and learning processes (Dohms et al., 2014). Educators ought to be competent, optimistic, enthusiastic, and certain they will proceed to flourish and instruct more successfully, as they are the focus of classroom transformation. They must have faith that they can involve their learners in education and enhance their educational results. They must be confident and pleased about their work and future in a way that brings back the worried-out passionately and physically (Derakhshan, 2021).

Educators carry out a variety of duties and activities, such as performing teaching and training activities, abiding by the laws of the civil service, conducting lessons consistently, performing their obligations, associating with school shareholders, carrying out responsibilities determined by the state, and accommodating to the variations they confront (Cetin, 2017). As stated by Tagay and Demir (2016), educators who occasionally face issues in carrying out these activities are affected by everything from job performance to health. Despite the problems they face, some educators proceed to work hard and vigorously, while others are helpless. Positive psychology (PP) scholars do not claim the absence of issues, but complement them with constructive themes as PP is a generic term for studying constructive feelings and character attributes, and this area of psychology emphasizes the research of personal abilities and capacities of achieving satisfaction and self-realization (Seligman, 2018; MacIntyre et al., 2019). Positive psychology emphasizes the cycles that play a part in the prospering and functioning of individuals, which emphasizes the virtues of citizens and institutions that inspire people to be better citizens (Wang et al., 2021). The function of feelings (affectivity) in different living situations is one of the main foundations of PP which also has such a function in language education (Dewaele et al., 2019). Furthermore, the impact of affectivity is greatly reflected in the other presumptions of PP: the strong points and the basic doctrines of enabling organizations (MacIntyre, 2016). Educators’ previous experiences and character attributes may play an important role in reflecting these practices. The fact that people behave differently in their career lives by assessing their lives from their viewpoints draws on the notion of well-being and as significant elements of teaching staff, language educators tolerate several destructive feelings and forces originating from their organizations, scholars, and coworkers, and language education itself is a career particularly replete with difficulties (Hiver and Dörnyei, 2015). Consequently, it is particularly essential to focus on the well-being of language educators. The concept of prosperity is usually considered when well-being is mentioned in PP (Kern et al., 2016). Well-being includes emotions of fulfillment and optimism as well as a feeling of authority and motive in life (Edmunds et al., 2013). Indeed, among the many institutions surveyed, the literature is focusing more and more on educator well-being. The teaching profession is a greatly sentimental one, involving great degrees of anxiety, and can lead to work dissatisfaction, mental illness, and diminished well-being (Keller et al., 2014). Moreover, the emotional well-being of educators is important not only to themselves but also to the learners, given that the educator plays a central role in the learners’ problems and learning of life (Jiang et al., 2016; Lee et al., 2016). In the English as a foreign language (EFL) context, educator well-being can also be threatened by several problems, including lack of language abilities, deficient language information, and lack of conviction (Talbot and Mercer, 2018). High-quality education is one of the most significant elements in educator effectiveness, and learners’ education and performance depend on educators’ inspirations, fulfillment, dedication, and constructive perspective (Bullough, 2011). These constructive perspectives and feelings assist with developing educators’ cognitive, social, and emotional assets, thereby helping them to deal with occupational demands and anxiety as well as contributing to their well-being (Frenzel et al., 2009).

Optimism is regarded as another issue that impacts well-being and lately, it has been at the center of scholars’ attention in educations aimed at determining in-school issues influencing learners’ academic success (Souri and Hasanirad, 2011). The concept of optimism can generally be characterized as the anticipation that individuals will have constructive experiences (Bressler et al., 2010). This optimistic standpoint mirrors a constructive and advantageous method to comprehend human behavior (e.g., in contrast to the notion of pessimism, which implies negative consequences) (Harpaz-Itay and Kaniel, 2012). Optimism is central to the field of PP, helping people lead successful and satisfying lives (Seligman and Csikszentmihalyi, 2000). Given that educators are the most significant element influencing learners’ performance, it is not surprising that educators’ scholastic optimism, which is believed to influence learners’ success, is the subject of study. Furthermore, scholastic optimism is considered an influential educator attribute of learners’ scholastic performance (Beard et al., 2010). It is generally defined as having constructive anticipations for the future, along with feelings, patience, and problem-solving; scholastic, athletic, military, professional, and political successes, health, and variously actualized in different research (Peterson, 2000). Theoretical methods such as personality characteristics, trait-related styles, and anticipated impacts are decisive for the actualization of well-being (Seligman, 2018). Studies show that optimism is closely related to personal resilience, life fulfillment, and resilience to deconstructive encounters (Segerstrom, 2007; Terzi, 2008). Most studies show that optimism is connected with a wide range of constructive components and well-being. For instance, Chang and Sanna (2001) point out that optimism is directly related to life satisfaction, constructive emotions, depressive signs, and deconstructive emotions. Similarly, optimism has a constructive association with coherence and aspiration and a deconstructive one with universalized stress disorder (Krok, 2015; Kelberer et al., 2018). Optimism has been connected to physical and mental health and functioning (Karademas, 2006). Furthermore, Liu et al. (2018) proved that optimism has a positive correlation with overall self-efficacy, life fulfillment, and constructive affection and a negative correlation with reticence and deconstructive affection.

Additionally, in the education sector, educators’ emotions are not a core problem and despite education focusing on emotions, very little research is led (Bakker, 2005). Also, the construct of optimism as an interactional event has been studied only in various situations like health and spiritual health care (Beach, 2003; Martinez, 2009; Wang and Guan, 2020), while research about teachers’ optimism in language learning context is not enough. Likewise, the effect of learners’ and educators’ optimism on students’ success is well proven in the literature (Lu, 2021); however, little is known about the role of teachers’ optimism and affectivity on their psychological well-being in the classroom. Therefore, this review aims to add to the prior literature by considering these concepts in language learning, especially about English learning.

## Review of Literature

### Positive Psychology

For the last 20 years, PP’s increasing popularity has triggered a strong step away from a sole emphasis on issues in general psychology. Positive psychology was created in the second half of the 20th century to lay greater importance on the constructive aspect of life (Dewaele et al., 2019; Wang and Guan, 2020; Budzińsk and Majchrzak, 2021; Wang et al., 2021). PP has existed for hundreds of centuries, going back to the notions of old scholars and devout leaders who conferred about personality, dignity, joy, and an upstanding community (Diener, 2009). Positive psychology is an experiential exploration of the lives of “ordinary” people to support their prosperity and growth (Seligman and Csikszentmihalyi, 2000).

### Affectivity

A person’s decision to learn a language and engage in classroom activities can be influenced by emotions, and both constructive and deconstructive types could have a significant impact on learners’ eagerness (Mendez Lopez and Pea Aguilar, 2013). It has been explained that deconstructive emotions like stress and unhappiness can promote education and could also be considered useful in the language education cycle. Language courses contain both detrimental and useful emotions (Benesch, 2017). Detrimental ones impede successful instruction and useful ones promote it. Moreover, because the teacher-student relationship is interactive, it contains personally meaningful material and singularity that are made simpler through educators’ relational and emotional attentiveness toward learners (Xie and Derakhshan, 2021). In addition, educators’ emotions are intertwined with their minds and urge, which are associated with their educational behavior. They are associated with other characteristics as well like educator character, burnout, emotion regulation, reflection, personal and professional life, as well as well-being (Fathi et al., 2020, 2021; Greenier et al., 2021).

Since educators’ feelings improve or impede the educators’ capability of instructing learners effectively, they have a significant impact on student’s scholastic life. Educators’ constructive feelings can have a significant impact on the quality of education and can improve the environment of the school as a whole, and deconstructive feelings can affect educators’ teaching and professional practice (Frenzel, 2014). Educators’ emotional capabilities promote an appealing educational setting and develop a sound and motivational ambiance for learners (Garner, 2010). Constructive feelings assist educators with creating innovative educational techniques that increase learners’ involvement and inspiration, while deconstructive feelings impair educators’ innovativeness and impact educational results (Frenzel et al., 2009).

### Well-Being

The word well-being is defined in many ways; in psychology, however, it is most commonly done utilizing individual well-being, constructive trait/personality strength, and lack of mental anxiety such as sadness, distress, and concern (Kern et al., 2015). Seligman (2018) characterized the concept of well-being in five main dimensions, all of which are part of a constructive continuum of mental well-being, called positive emotions, engagement, relations, meaning, and achievement, abbreviated as PERMA. Positive emotions such as joy, optimism, and happiness are all in a pleasurable continuum of emotional conditions that can act as a sign of wealth, even though they can prosper, be taught, and be enhanced (Fredrickson, 2001). Engagement is commonly referred to as a form of flow or profound engagement that is intended to be a natural motivator when completing a task and it refers to the point of involvement that shows the level of enthusiasm and interest for issues such as renovation or well-being (Rajabi and Ghezelsefloo, 2020; Derakhshan, 2021). Contributors to well-being throughout life include determining objectives, monitoring, and achieving (Heckhausen et al., 2010). Positive relationships imply a sense of being socially integrated, recognized, and strengthened by others, and a sense of enjoying one’s social connections. Social support is associated with mental and physical well-being outcomes as well as emotional well-being (Wang and Guan, 2020; Greenier et al., 2021). Well-being is guaranteed by building meaningful connections with peers, family, and colleagues. A sense of belonging can result from sympathetic or deconstructive relationships like intimate ones with friends and family, or poor ones with them (Sandstrom and Dunn, 2014). Meaning is the idea that a person’s life involves persistence and a route in the lifecycle as well as a sense of belonging to a superior entity. It is related to well-being outcomes and positive emotions of different age ranges. As for achievement, it is generally associated with determining objectives, growing, and possessing the capability of accomplishment, and so endeavoring for well-being (Croom, 2015; Kansky and Diener, 2017).

The notion of well-being in constructive psychological research is an intricate concept involving two major methods: personal well-being and mental well-being (Diener et al., 2010). The former type of well-being is shown by hedonic standards while the latter type of well-being is shown by eudemonic ones (Kallay and Rus, 2014). Well-being means the best of all the goods that people attain through actions and emotions that are in harmony with their true selves. Therefore, the eudemonic method constitutes mental well-being in the sense of significance, maximum functioning, and self-realization (Garg and Rastogi, 2009). Psychological well-being refers to the global emotional circumstances and copes with the capability of individual psychology and it has been considered as dynamic administration that assists peoples to come across their crises and problems, come across and even solve difficulties of their lives, and accomplish the objectives (McDool et al., 2020).

Psychological well-being (PWB) can be characterized as a mix of constructive emotional conditions like joy, pleasure, and working with maximum affectivity in personal as well as social lives (Deci and Ryan, 2008). Psychological well-being involves determining the continuous well-being of an individual; fulfillment with an individual’s physical and mental health (the hedonic viewpoint) and how this is associated with mental elements like life and career fulfillment (the eudemonics viewpoint) (Garg and Rastogi, 2009). Psychological well-being is a complex concept and is affected by numerous elements and the self-determination theory by Deci and Ryan (2008) offers an all-encompassing description of the intrinsic inspirations and their function in PWB development. Based on this model, three fundamental demands must be met to promote personal health and well-being: autonomy, competence, and relatedness. Autonomy alludes to the inclination of being causal and autonomous actors who function in a balanced manner with the consolidated self. Competence is an experience of an action performed effectively. Ultimately, a global demand to associate and connect with others, and experience taking care of others, is known as relatedness (Deci and Ryan, 2008).

### Optimism

The attribute of optimism is one of the two most broadly studied notions inside PP that investigates and describes ideal settings (Seligman, 2018). Positive psychologists distinguish circumstances in which people prosper and grow by assessing constructive feelings (optimism in particular), attributes, and organizations (Hoy et al., 2008). Optimism is characterized as a static personality attribute connected to constructive anticipations regarding occurrences in the future. While pessimists anticipate deconstructive results, optimists anticipate constructive ones (Carver et al., 2010). Likewise, optimism possesses a cognitive component related to inspiration. Optimists demonstrate endeavor whereas pessimists keep well away from making efforts and Optimists are better at dealing with difficulties, and optimism has been found to be a better source of psychological health. There is also a linear relationship between elevated optimism and elevated individual well-being (Carver et al., 2010). Optimism, or being optimistic about the future, can be characterized as the expectation or conviction that good will occur in significant areas of life (Scheier and Carver, 2009). Optimistic people are inclined to be less pessimistic and have constructive and advantageous anticipations regarding their future. Optimism allows individuals to proceed with their constructive anticipations as opposed to emphasizing issues since optimists usually possess a sense of certainty and believe that challenges can be dealt with effectively and that objectives in life can be attained through different means (Scheier and Carver, 2009). Moreover, optimism is positively correlated with coherence and a sense of hope and is negatively correlated with universalized stress disorder (Kelberer et al., 2018).

Optimism, as mental power, appears to be a solid indicator of SWB and optimism, or having optimistic thoughts regarding what is to come, maybe characterized as the anticipation or conviction that pleasant occurrences will take place in significant areas of life (Scheier and Carver, 2009). Optimistic people tend to be less pessimistic and have constructive and advantageous anticipations about their future. It allows individuals to keep on having constructive anticipations as opposed to highlighting issues (Peterson, 2000) this is due to the fact that optimists usually possess the confidence and believe that they can successfully overcome challenges and attain their objectives in life through various means (Scheier and Carver, 2009).

An important element deciding educators’ effectiveness and learners’ results is educators’ scholastic optimism (Hoy et al., 2008). Scholastic optimism initially characterized as a school cultural trait, recognizes the interrelationship between the way people attain knowledge and activities and their social setting. It is designed as a multi-faceted concept, including cognitive, behavioral, and attitudinal elements, that pervades the educator’s daily routines, develops a constructive educational setting, and promotes sustainability. Therefore, principles and emotional personalities affect the way educators comprehend their roles and accountabilities and accomplish their proficient repetitions. Educators’ scholastic optimism is a three-element concept of educator self-assurance, educator scholastic emphasis, and educator faith in learners and guardians (Hoy et al., 2006). Educators’ scholastic optimism incorporates their constructive convictions regarding themselves, regarding their learners and guardians, and this constructive vitality goes further to highlight education for the learners invigorated by the conviction that they will triumph (Hoy et al., 2006).

As stated by Tschannen-Moran and Hoy (2001), self-efficacy is central to all educators’ clarifications regarding the decision to remain in, move from, or leave their schools. It was mainly dependent on their awareness and convictions about being successful with their learners. How successful educators believe they are when it comes to assisting learners with success is known as educators’ efficacy, and this has an influence on learners’ education. Self-efficacy has also been demonstrated to have a direct impact on performance, which corresponds to overall capability. Teachers who hold high levels of self-efficacy often adopt suitable educational methods and techniques, leading to increased student involvement and scholastic success (Ipek et al., 2018). Effective educators should be capable of building relationships of trust with their learners and guardians. Trust relationships incorporate mercy, credibility, ability, integrity, and openness (Tschannen-Moran and Hoy, 2001; Han and Wang, 2021). Generally, educators need to trust their learners’ willingness to learn, their ability to understand notions, and their integrity. Like educators’ sense of efficacy, educators have great expectations for trusted learners and count on the help of their guardians. Optimism is a forceful topic that combines assurance, faith, and scholastic emphasis since each of these factors has a sense of what is possible. Optimism means contemplating the future. Furthermore, it is a constructive feeling, a constructive power associated with patience and individual control, and is known to expand and promote an individual’s cognitive and behavioral repository (Peterson, 2000). As declared by Rand (2009), optimism is conventionally regarded as fixed to the idea of goals and is thus closely associated with the notion of hope. Nonetheless, while hope solely refers to a belief in one’s ability to find different paths for the achievement of wanted objectives, optimism may be described as an anticipation of achievement in life when faced with misfortune and damages that is uncontrollable (Rand, 2009).

## Implications and Future Directions

This review has significant suggestions for educators from various academic organizations, school leaders, and academic psychologists. It aims to influence academic and organizational psychology and also it adds to the literature intending to clarify the significance of educators’ constructive emotions in the teaching space especially in language classroom. By instructing and allowing learners to learn in the best possible way, good educators are capable of making a meaningful difference in their community, and their emotions are a strong means of facilitating or impeding education. According to the upshots of the present review, there is an urgent need for academic organizations to pay close attention to educators’ constructive emotions like optimism. Indeed, educators’ eagerness to instruct as well as the people with whom they associate is influenced by educator optimism and optimistic educators generally look at the positive side and focus on the constructive attributes of learners, schools, and societies.

Moreover, presenting consistent training for educators in the field of well-being and constructive emotions is crucial in learning context, as well. EFL Teachers’ emotions about the workplace depend largely on the setting they are in and how well they are treated. The optimism of EFL teachers was also found to be in relation to their PWB. Scholastically optimistic educators are good at motivating their students and when teachers are optimistic, they place great value on scholastic-related matters and have great expectations for their learners’ success and achievement. Besides, scholastic optimism is educators’ constructive attitude that they can accomplish their role by focusing on education and learning, relying on guardians’ and learners’ involvement, and having faith in their ability and efficiency to solve issues and conquer failures; therefore, they can work well and enhance school success and learner performance. Educators can also have a significant part in enhancing interactive relationships between coworkers and school leaders. Content, honest, and emotionally stable educators are optimistic about learners, coworkers, and school leaders and when educators are optimistic, they have faith in their educational skills and guardians’ support for education.

In addition, this review is significant for EFL teachers in the language learning context as they should be conscious of their significant function. Educators are the core of any educational institution, and their well-being should be paramount in the instructional setting to enhance students’ capability and to inspire and motivate them to take part in-class activities and educators with high levels of well-being can proactively address vocational demands and successfully control the emotional circumstances that arise when working with learners of various ages. The foundation for developing connections with learners is educators’ well-being that it offers a psychologically safe, learning-oriented class so they need to build a secure, encouraging, and inspiring class where learners are truly listened to. Educators and learners must be aware of the connections and the levels of attachments that they share. Educators must grasp that their well-being has a spillover influence on their learners, thus to carry out their role successfully, they need to build their well-being skills, as well and they must have a dominant and collaborative educational technique that ensures learners are free and disciplined when needed. Moreover, they must frequently take part in training and individual growth to acquire the information, abilities, and capacities to successfully control and create learners’ well-being in the class (Poulou, 2016). EFL Teachers’ emotions and optimism are among the constructs anticipated to be important indicators of well-being and it is crucial and helpful for educators, and the school government’s presentation and well-being. Emotions are transmittable, and the more educators like their workplace, the more inspired they are when arranging and instructing (Bakker, 2005). Optimism is essentially viewed as having constructive anticipations for the future. People with constructive anticipations are likely to be more adaptable, more open to alternative thinking, and more constructive about challenges than those with deconstructive thoughts. From the well-being approach in general and PWB in particular, an individual has low levels of anxiety, fault, pressure, irritation, and aggression and he/she is moderately optimistic and free of apprehensions. Educators’ optimism is significant since they are exemplars for learners and their perspectives have an immense effect on their lives. Educators’ optimistic perspective and constructive character attributes can correspondingly have a positive impact on a learner’s life and it helps them see the cup half full even on difficult days and deal with them successfully; hence, it was essential to distinguish the constructive dimensions of school and university educators. The comprehension of educators’ beliefs and their degrees of educational optimism is key information for educator tutors to improve and cultivate pre-service platforms that will arrange knowledgeable educators arranged to fairly meet the needs of learners. EFL Teacher tutors should try to improve and nurture educators’ optimism through highlighting strong provision of various types of knowledge such as content, pedagogical, and even technical knowledge.

Besides, it can be stated that educators with positive feelings only emphasize the constructive traits of their learners and the constructive qualities of their classes, public, and schools. The school manager should be careful about the classroom context and undeniably, classrooms that nurture and even boost positive feelings among educators and learners through the progressions of training and scholarship are extremely likely to arrange for the greatest conditions for encouraging learners’ educational growth and success (Yan et al., 2011). Teacher education and training courses must recognize the emotional aspects of educators, help them recognize the professional pertinence and worth of their emotional experiences, and help them reflect on such experiences critically. The school manager should increase levels of optimism in teachers to build a constructive and healthy school environment. These optimistic perspectives propose that learners’ performance improves when educators are optimistic about their capability of supporting and engaging learners. These perspectives further propose that learners’ performance improves when educators have a constructive mentality and anticipate constructive results.

This field of study is also useful for supervisors who solely monitor the cycle of attracting educators to perform activities and regard educators as a means to achieve objectives so that they can come up with the right judgments on their own and decide on how to carry out tasks. This builds inspiration and scholastic optimism among educators and consequently triggers their well-being. Educator trainers must be aware that educator obligations are not merely the provision of material or educational knowledge. Indeed, educators are obligated to create influential connections, develop connections of trust between themselves and learners, and establish a pleasant educational setting. Furthermore, materials developers can make materials that reflect the feelings of educators, and develop assignments and activities that assist educators with relieving stress and the workload in the workplace, and help them enjoy the moment and promoting their well-being. Further research can be led to explore PP-centered themes in education so more studies and surveys can be carried out on various constructive feelings in instruction, and the factors that elevate educators’ well-being may be investigated. These endeavors play a direct part in educators’ well-being and indirectly enhance their achievement. Therefore, enthusiastic researchers are suggested to work on the constructs presented in this manuscript about other variables like self-efficacy, hopefulness, joy, and relational communication behaviors like intelligibility, credibility, or immediacy. Succinctly, it is likewise required to relate EFL teachers’ optimism to learners’ achievement and investigate and scrutinize this association in further studies.

## Author Contributions

The author confirms being the sole contributor of this work and has approved it for publication.

## Conflict of Interest

The author declares that the research was conducted in the absence of any commercial or financial relationships that could be construed as a potential conflict of interest.

## Publisher’s Note

All claims expressed in this article are solely those of the authors and do not necessarily represent those of their affiliated organizations, or those of the publisher, the editors and the reviewers. Any product that may be evaluated in this article, or claim that may be made by its manufacturer, is not guaranteed or endorsed by the publisher.
